# Chemical Composition of Two Different Lavender Essential Oils and Their Effect on Facial Skin Microbiota

**DOI:** 10.3390/molecules24183270

**Published:** 2019-09-08

**Authors:** Marietta Białoń, Teresa Krzyśko-Łupicka, Ewa Nowakowska-Bogdan, Piotr P. Wieczorek

**Affiliations:** 1 Faculty of Chemistry, University of Opole, Oleska 48, 45-052 Opole, Poland; 2 Independent Department of Biotechnology and Molecular Biology, Faculty of Natural and Technical Science, University of Opole, Kominka 6A, 45-035 Opole, Poland; 3 The Institute of Heavy Organic Synthesis “Blachownia”, Energetyków 9, 47-225 Kędzierzyn-Koźle, Poland

**Keywords:** facial skin microbiota, gas chromatography with mass spectrometry, lavender essential oil

## Abstract

Lavender oil is one of the most valuable aromatherapy oils, its anti-bacterial and anti-fungal activities can be explained by main components such as linalool, linalyl acetate, lavandulol, geraniol, or eucalyptol. The aim of the study was to assess the anti-microbial effects of two different lavender oils on a mixed microbiota from facial skin. The commercial lavender oil and essential lavender oil from the Crimean Peninsula, whose chemical composition and activity are yet to be published, were used. Both oils were analysed by gas chromatography coupled to mass spectrometry. The composition and properties of studied oils were significantly different. The commercial ETJA lavender oil contained 10% more linalool and linalyl acetate than the Crimean lavender oil. Both oils also had different effects on the mixed facial skin microbiota. The Gram-positive bacilli were more sensitive to ETJA lavender oil, and Gram-negative bacilli were more sensitive to Crimean lavender oil. However, neither of the tested oils inhibited the growth of Gram-positive cocci. The tested lavender oils decreased the cell number of the mixed microbiota from facial skin, but ETJA oil showed higher efficiency, probably because it contains higher concentrations of monoterpenoids and monoterpenes than Crimean lavender oil does.

## 1. Introduction

The generic name “lavender” dates back to ancient times and derives from the Latin word *lavare*, which means washing and bathing. According to Romans, not only the aromatic qualities but also antiseptic properties were important [1]. Therefore, lavender oil was used as a panacea even in the case of wounds associated with tissue loss [2].

Today, as a preservative and skin regenerator, it is widely used in the cosmetic industry to produce safe tonics, lotions, creams, shampoos, conditioners, shower gels, and soaps by specialist cosmetic companies, such as Dr. Beta-Pollena Aroma, Farmona Organique, Sanoflore, and Yves Rocher.

Because of the mild climate, adequate sunshine, alkaline soil, and natural wind protection, lavender is naturally present in Mediterranean countries. 

Lavender belongs to the *Labiatae* family, which includes ~30 species of *Lavandula*. However, only three species with lavender fragrance are of industrial importance. These are as follows [3]:

1. Narrow-leaved lavender (real, medical) *Lavandula officinalis* Chaix, syn. L. *vera* DC, *L. angustifolia* Mill.;
2. Broad-leaved lavender (spike lavender) *Lavandula latifolia* Vill. (Syn. *L. spica* DC);
3. Lavandin, a hybrid of the two preceding species.

In the cosmetics industry, the most popular essential oils are those derived from these plants. Nevertheless, both their odour and chemical composition are determined by a number of factors: plant species or varieties, climatic conditions, growth method and conditions, harvesting, transport, storage, and oil preparation techniques. Lavender oil obtained from *L. angustifolia* is the most valuable and the most expensive because its efficiency is two-fold lower than that of spike oil, and four-fold lower than that of lavandin oil [4]. Therefore, lavender oil can be falsified with cheaper oils (lavandin or spike). Sometimes, synthetic products such as linalyl acetate are added [4].

Although the main active ingredients are monoterpenes (linalool, linalyl acetate, lavandulol, geraniol, bornyl acetate, borneol, terpineol, and eucalyptol or lavandulyl acetate), these oils may have different anti-bacterial and anti-fungal effects, depending on their chemical composition [5,6]. A high and almost equal content of linalool and linalyl acetate (a ratio above one) is required for good anti-microbial properties of lavender essential oil [5,6]. The high concentration of lavandulol and its acetate is also desirable, giving the oil a rosaceous, sharp floral aroma. Good anti-microbial activity requires that the ratio of the content of the sum of linalyl acetate with linalool to the content of terpinen-4-ol in lavender essential oil is more than 13 [3,4]; moreover, this ratio can help to determine the type of oil and its applicability, as presented in Table 1 [7,8,9]. On the other hand, high concentrations of ocimene, cineole, camphor, or terpinen-4-ol adversely affect the quality of this oil [3,4]. The chemical composition of different lavender species depends on the geographic region of origin (Table 1 and Table 2). On the basis of the presented observations, it is obvious that *L. angustifolia*, *Lavandula stoechas*, and *Lavandula dentata* contain large amounts of eucalyptol and camphor (Table 1 and Table 2). It should also be noted that lavender oil from Brazil [10] contains borneol at a concentration of 22.4%, which is much higher than that in other *L. angustifolia* oils (Table 1). Although *L. abrialis* oil from France [11] and *L. bipinnata* from Algeria [12] contain large amounts of camphor, they do not contain eucalyptol (Table 2).

Because each lavender oil has a quantitatively and qualitatively distinct profile of chemical compounds, it is necessary to determine the quantity and identity of its individual components (Table 1 and Table 2). These data will allow researchers to determine the effects of essential lavender oils on the autochthonous microbiota of the skin. From the literature [23,24], it is known that the chemical composition of oils and their macerates has a decisive influence on their microbiological properties.

Lavender essential oil contains several anti-microbial compounds, such as eucalyptol, linalool, terpinen-4-ol, and *α*-terpineol. Among them, linalool was demonstrated to be the strongest active ingredient against a wide range of microorganisms [8]. Borneol and eucalyptol were identified also as the main compounds in the many essential oils exhibiting anti-parasitic activity [10]. Terpinen-4-ol, *α*-pinene, *β*-pinene, 1,8-cineol, linalool, and 4-terpineol also showed high anti-fungal activity against Gram-positive and Gram-negative strains [25,26]. Linalool and linalyl acetate have local anaesthetic effects, proven in animal tests (in vivo and in vitro) [25]. Various monoterpenoids, such as α-terpineol, terpinen-4-ol, eucalyptol, and linalool, have antiviral activity against influenza strains [26]. Eucalyptol, terpinen-4-ol, thymol, and carvacrol also have extensive anti-inflammatory effects [26].

Composition of the facial skin microbiota varies and depends on many factors, such as proper hygiene, state of health, oiliness, skin hydration, pH, local temperature, and the reduction potential [27]. The skin microbiota contains persistent indigenous microorganisms (so-called residents) that are located on its surface for almost the entire lifespan of the individual, including transitory microorganisms from the environment, animals, food, or water. The microbiota of adult skin [27,28,29] is mainly formed by Gram-positive cocci (*Staphylococcus epidermidis*, *S. haemolyticus*, *S. hominis*, *S. aureus* (carrier), *Enterococcus faecalis*, *Micrococcus* spp., and *Streptococcus*), Gram-positive bacilli (*Corynebacterium* spp., *Propionibacterium acnes*, *P. granulosum*, *P. avidum*, and *Bacillus* spp.), Gram-negative bacilli (*Acinetobacter spp.* and *Escherichia coli*), and yeast-like fungi (*Pityrosporum ovale* and *Candida* spp.). Cosmetics containing active anti-bacterial substances of natural origin (essential oils) help to control the growth of microorganisms and additionally have a beneficial effect on the processes taking place on the surface and in the skin. Essential oils accelerate the regeneration and development of skin cells, and, for this reason, the skin becomes stronger and regenerates faster by supporting the processes of granulation of a wounded epidermis [30]. As a result, skin ageing processes are delayed.

The chemical composition and anti-microbial activity of different lavender oils depend on the geographic region of origin, which is why the aim of the present study was to analyse the correlation of the chemical composition of two lavender oils of different origins with their anti-microbial effects on the mixed microbiota of facial skin and on dominant bacterial isolates extracted from the surface of facial skin. The chemical composition and activity of essential lavender oil from the Crimean Peninsula are yet to be published.

## 2. Results

### 2.1. Chemical Analysis

A complex chromatogram was obtained as a result of this analysis, where we identified 101 compounds. Among these identified components, there were 64 oil compounds that were already described in the literature and 37 other compounds, which are yet to be reported (Table 3).

Based on the results, it was found that Crimean lavender oil contains several-fold larger quantities of monoterpenes such as 3-carene, *o*-cymene, or bicyclic sesquiterpenes (bergamotene isomers, caryophyllene, or *γ*-cadinene) as compared with the literature data. In the extract of Crimean lavender, *p*-cymene-1-ol, 3-octanone, and terpenes were detected at much smaller concentrations, e.g., sabinene or ocimene and sesquiterpene germacrene D.

Analysis of hexane solutions of lavender essential oil was performed too (on the SupelcoWAX column), and the average retention parameters and peak areas are listed in Table 3. Less complex chromatograms and worse separation of the sample components were obtained in this assay; specifically, the peaks corresponding to the main components of oil were close to one another and were not completely separated.

GC–MS analysis on the SupelcoWAX column allowed us to identify 50 compounds contained in Crimean lavender essential oil. Among these compounds, 42 were already described in the literature. Three components of lavender oil—which were already described in the literature—could not be identified by means of the HP-5MS column but could be identified using the SupelcoWAX column. These compounds included *γ*-terpinene, octene-3-yl acetate, and norborneol acetate.

Furthermore, eight previously undescribed components were identified, including the five already identified via the HP-5MS column. In addition, the presence of three small peaks corresponding to benzoic acid, butyric acid, zingiberene, and 2-phenylethanol was detected.

**Figure 1:**
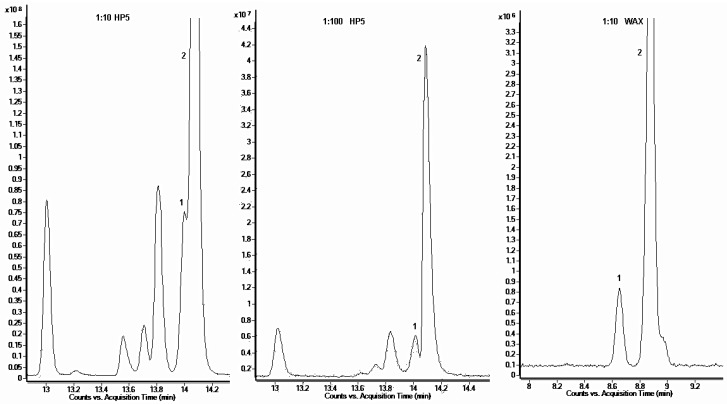
Comparison of limonene (1) and eucalyptol (2) peak separation, between the HP-5MS column at a dilution of 1:10 (*v*/*v*) or 1:100 (*v*/*v*) and the SupelcoWAX^TM^ 10 column at dilution 1:10 (*v*/*v*).

Moreover, sharp, clear-cut, and completely separated peaks of limonene (1) and eucalyptol (2) for 1:10 dilutions were obtained using this column. They could not be analysed by means of the HP-5MS column. In the case of samples with the dilution of 1:10, overlapping limonene (1) and eucalyptol (2) peaks were observed. Some improvement of separation of these components of lavender oil was achieved by greater dilution, but only the use of polar columns yielded satisfactory results. The comparison of separation of these two terpenes at different dilutions on both columns is shown in Figure 1.

From the presented data, it can be concluded that the separation of Crimean lavender oil on the polar column was not satisfactory; better results were obtained on the non-polar column. Therefore, only this column was employed to determine the chemical composition of ETJA lavender oil.

On the basis of the conducted studies, it was demonstrated that the tested lavender oils differ in chemical composition and anti-microbial activity both quantitatively and qualitatively. In ETJA oil (Table 3), 33 components were identified, including 28 already described in the literature, as well as five compounds not described previously (2-carene, cyclofenchene, *β*-copaene, 2-bornanone, and isoborneol).

ETJA lavender oil turned out to contain higher concentrations of linalool (41.8%), linalyl acetate (32.7%), and limonene (19.0%), whereas Crimean lavender oil contained linalool (34.1% according to HP-5MS column analysis or 52.7% in accordance with the SupelcoWAX column analysis), linalyl acetate (23.3% according to the HP-5MS column analysis or 36.6% according to the SupelcoWAX column analysis), and eucalyptol (5.0% in accordance with the HP-5MS column analysis or 1.7% judging by the SupelcoWAX column analysis; Table 3). ETJA lavender oil was composed mainly of monoterpenoids (76.7%) and monoterpenes (22.7%), whereas Crimean lavender oil was found to be composed mainly of monoterpenoids (80.1% according to the HP-5MS column analysis or 95.1% in accordance with the SupelcoWAX column analysis), much less monoterpenes (5.8% according to the HP-5MS column analysis or 1.6% judging by the SupelcoWAX column analysis), and some sesquiterpenes (8.0% in accordance with the HP-5MS column analysis or 2.3% judging by the SupelcoWAX column analysis; Table 4). 

### 2.2. Biological Analysis

**Figure 2:**
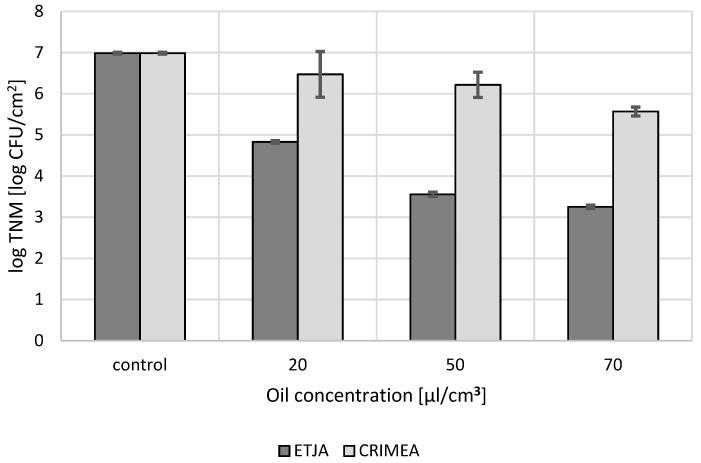
The influence of concentrations of the lavender oils under study on the number of microbiota cells from facial skin.

The effect of lavender oils on the mixed microbiota of the face skin without signs of lesions depended on the origin of the oil and the concentration used. ETJA lavender oil at all concentrations tested reduced the number of skin microbial cells 1000–10,000-fold, compared to the control. The strongest microbial cell number reduction was observed after application of 70 µL/cm^3^ oil and slightly less at 50 µL/cm^3^ (Figure 2). On the other hand, Crimean lavender oil exerted much weaker anti-microbial activity, and only at the highest concentration did it suppress the growth of the microbiota hundred-fold (Figure 2).

Lavender oils, depending on their origin, also had a different influence on the qualitative changes in the facial skin microbiota. In the presence of the highest concentration of ETJA lavender oil tested, bacteria of the following species survived: *Micrococcus luteus*, *E. coli*, *Staphylococcus warneri*, and *Enterococcus faecium*. Crimean lavender oil, however, did not inhibit the growth of *Bacillus* (*B. cereus*, *B. subtilis*, and *B. mycoides*), *Corynebacterium* spp., *E. faecium*, and *S.* *warneri*. The most sensitive to ETJA lavender oil were Gram-positive bacilli, and Gram-negative bacilli were the most sensitive to Crimean lavender oil. On the other hand, none of the tested oils inhibited the growth of Gram-positive cocci.

**Figure 3:**
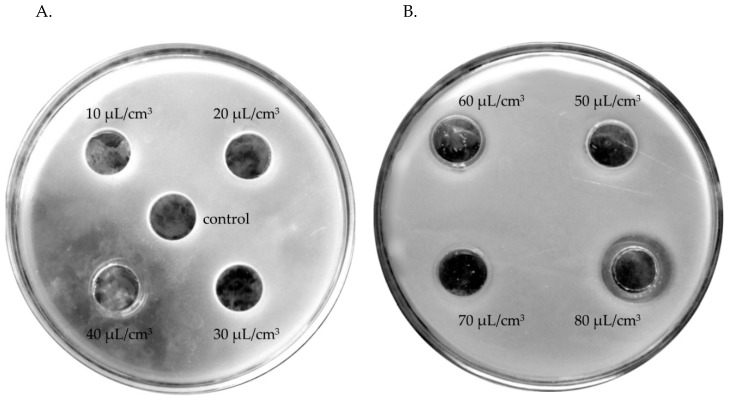
Zones of growth inhibition of a *Bacillus cereus* isolate in the presence of tested concentrations (10–80 μL/cm^3^) of lavender oils: (**A**) ETJA, 40 μL/cm^3^; (**B**) Crimean lavender oil, 80 μL/cm^3^.

Therefore, an attempt was made to determine those oil concentrations which would effectively decrease the growth of individual isolates. Inhibitory effects on the growth of bacterial isolates that survived in a mixed microbial population from facial skin were exerted by the tested oils only at concentrations between 40 and 80 μL/cm^3^ and growth inhibition zones between 12.5 and 44 mm, with higher effectiveness of oils at the highest concentrations. ETJA lavender oil was more effective because it limited the growth of most bacteria under study, including *Bacillus*. Neither of the oils tested inhibited the growth of *E. faecium* (Table 5, Figure 3).

**Figure 4:**
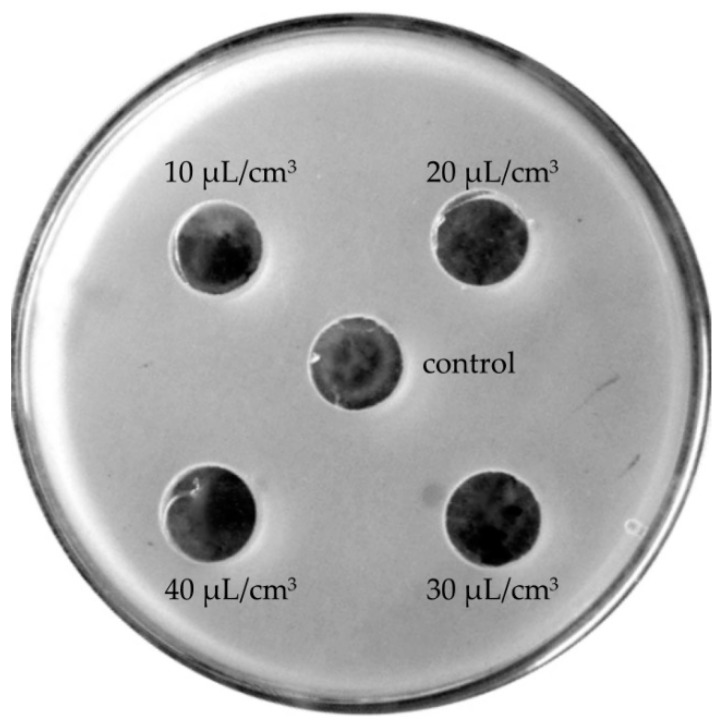
Neutral effects of lavender oils on the growth of the bacterial species *Enterococcus faecium*.

**Figure 5:**
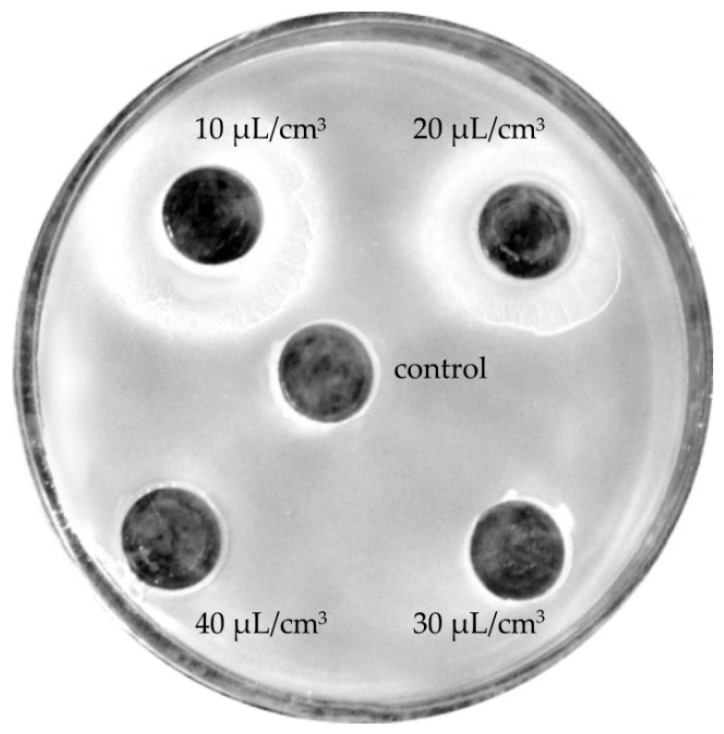
Stimulation of *Bacillus mycoides* growth in the presence of a low concentration (10 or 20 μL/cm^3^) of Crimean lavender oil.

At lower concentrations (10–40 μL/cm^3^), lavender oils manifested neutral effects (Figure 4) or, as in the case of Crimean lavender oil, stimulated bacterial growth (Figure 5).

To sum up, the tested lavender oils reduced the mixed population of microbes from facial skin, but ETJA lavender oil with higher amounts of monoterpenoids (linalool and linalyl acetate) and monoterpenes (limonene) was characterised by higher effectiveness than Crimean lavender oil.

## 3. Discussion

Shortly after birth, the human skin is immediately colonised by microorganisms, such as bacteria, yeasts, fungi, and viruses. Nonetheless, the composition of the skin microbiota varies quantitatively and qualitatively, and it depends on humidity, temperature, pH, and body area [27]. A child’s skin is mainly colonised by bacteria of genus *Staphylococcus*, *Enterococcus*, *Corynebacterium*, and *Escherichia*, and, in the teenage period, by *Sarcina*. At an elderly age, however, an increase in the number of fungal cells is observed, mainly *Candida albicans* yeast [27,28,29].

The challenge in skincare is oily skin because it has to be properly cleaned and moisturised, but comedogenic agents (which block sebaceous glands, resulting in blackheads) cannot be used [27,31,32]; therefore, biological substances effective at low concentrations are sought for care for this type of skin. Lavender oil is a strong antiseptic. Therefore, it is an additive to pharmaceuticals (salve and lotions for hard-to-heal wounds, eczema, and anti-rheumatic preparations), as well as cosmetics. It is used in mouth, throat, upper respiratory tract, and lung infectious diseases, as well as in dermatology to treat difficult-to-heal wounds, ulcers, and burns, and in cosmetology. However, the anti-microbial effect of lavender oil depends on the species and the variety of lavender from which it is obtained. Cavanagh and Wilkinson [33] and Sienkiewicz et al. [34] showed that the anti-microbial activity of essential oils depends on their chemical composition. According to literature data, *Lavandula angustifolia* oil has the most variable chemical composition. Bulgarian lavender oil contains ocimene (6.8–7.7%), linalool (30–34%), and linalyl acetate (35–38%), while it does not contain lavandulol and lavandulol acetate. The main ingredients in oils from China and India were linalool, linalool acetate, and lavandulol, all found in various amounts; however, ocimene was not identified [14,35]. Adaszyńska et al. [36] showed that the highest content of linalool was found in essential oils from the variety “Lavender Lady” and “Elegance Purple” (23.9% and 22.4%). At the same time, these oils contained small amounts of *cis*-*β*-ocimene. The best anti-bacterial properties against *S. aureus* and *Pseudomonas aeruginosa* were found in oils obtained from varieties “Blue River” and “Munstead”. Essential oil obtained from *Lavandula angustifolia* Mill. has strong bactericidal properties against methicillin-resistant *Staphylococcus aureus* (MRSA) and vancomycin-resistant *Enterococcus* sp. (VRE) [33]. Essential oil from *Lavandula heterophylla* “Avonview” inhibits growth of *Streptococcus pyogenes*, *Enterobacter aerogenes*, *Staphylococcus aureus* MRSA, *Pseudomonas aeruginosa*, *Citrobacter freundii*, *Proteus vulgaris*, *Escherichia coli* VRE, *Shigella sonnei*, and *Propionibacterium acnes* [33,37]. A suitable substance may be lavender oil, which, according to the manufacturer, can be added even at a concentration of ~5% to cosmetic preparations and pharmaceuticals. At this concentration, lavender oil has a strong effect on some types of skin without irritation; however, more sensitive skin requires preparations with lower lavender oil content, and, during a disease with relevant symptoms, preparations with a higher concentration of lavender oil.

The scientific literature mainly contains the results of studies on the effects of lavender oils (obtained from many varieties of lavender by various research techniques) on selected individual isolates of microorganisms. Sabara and Kunicka-Styczyńska [38] reported that lavender oil (from *Lavandula angustifolia*) at concentrations of 100–200 µL/cm^3^ inhibited the growth of all tested microorganisms—*E. coli*, *Bacillus subtilis*, *Candida mycoderma*, and *Aspergillus niger*—and the inhibitory effect on *Bacillus* bacteria and *Aspergillus* fungi growth was obtained at a 10-fold lower dose (10 µL/mL). Roller et al. [39], when comparing the anti-microbial efficacy of several lavender oils (from different varieties of the plant), tested them individually, as well as in mixtures, against methicillin-resistant and non-methicillin-resistant *S. aureus* and noted that the best anti-microbial effects were obtained by combining several oils. In other studies, lavender oil at concentrations below 2000 ppm (parts per million) was less active against bacteria of the genera *Bacillus*, *Lactobacillus*, *Clostridium*, and *Bifidobacterium* [40].

The obtained results showed a significant reduction in the number of microbial cells in the mixed population from the skin at the dose of 50 µL/cm^3^ lavender oil, but the most effective was lavender oil at the concentration of 70 µL/cm^3^, although no complete inhibition of the growth of the mixed microbiota from the skin was observed. 

After application of ETJA lavender oil to the mixed microbiota from the skin, bacteria *M. luteus*, *E. coli*, *S. warneri*, and *E. faecium* survived, whereas, after the application of Crimean lavender oil, *Bacillus* (*B. cereus*, *B. subtilis*, and *B. mycoides*), *Corynebacterium* sp., *E. faecium*, and *S. warneri* survived. Only oils at concentrations between 40 and 80 μL/cm^3^ inhibited the growth of individual bacterial isolates, whereby oils used at the highest concentrations showed higher effectiveness. ETJA lavender oil inhibited the growth of most bacteria tested, including *Bacillus*, but neither oil inhibited the growth of *E. faecium*.

The most sensitive to ETJA lavender oil were Gram-positive bacilli, and Gram-negative bacilli were the most sensitive to Crimean lavender oil. On the other hand, neither of the tested oils inhibited the growth of Gram-positive cocci.

The essence of the effect of lavender oils on skin microbiota depends on the quantitative and qualitative chemical composition. Essential oils have an affinity for lipid cell structures; therefore, they destroy the cell wall and membranes of bacteria, mainly Gram-positive ones (less often Gram-negative) and fungi, and, as a consequence, there is leakage and coagulation of the cytoplasm. In addition, lavender oils inhibit the synthesis of RNA, DNA, proteins, and polysaccharides, while, in fungi, they act as anti-mycotics and inhibit the production of enzymes [40].

Monoterpenes, especially linalool, have an anti-microbial effect on bacteria. The mechanism of action consists of disturbing the lipid structure of cell membranes and increasing the permeability of these membranes to monoterpenes, which—by penetrating bacterial cells—block their metabolism, thereby leading to cell death [41].

According to the literature, the spectrum of action of lavender oil is broad because it has anti-viral and anti-fungal properties, in addition to bactericidal activity. Studies conducted by Minami et al. [42] revealed that narrow-leaved lavender (*L. latifolia*) spike oil at a concentration of 1% suppressed the replication of a herpes virus in vitro. According to those researchers, this outcome can be explained by the impact of oil components on the areola and virus glycoprotein [42].

Our study indicates that the main components of the tested Crimean lavender oil are similar to those of oils extracted from two species of lavender: *L. angustifolia* from Australia [13] and *Lavandin abrialis* from France [11]. Much greater differences from the literature data were observed here in the components present in the tested oils in quantities not exceeding 1%. Crimean lavender oil is characterised by several-fold higher concentration of terpenes such as 3-carene, *o*-cymene, caryophyllene, and bergamotene as compared to other lavender oils. Nonetheless, sabinene, ocimene, and germacrene D were present in much smaller quantities. In addition, we were able to identify other terpene compounds not yet reported as components of lavender oil. These include terpene alcohols (geraniol, farnesol, santalol, isopulegol, and cubenol), terpene aldehydes (citral), and terpene ketones (verbenone, cinerone, and eucarvone).

## 4. Materials and Methods

### 4.1. Materials

The research experiments consisted of two oils: commercial lavender essential oil (*Lavandula angustifolia* Oil) from ETJA (produced by ETJA, Elbląg, Poland) and Crimean lavender oil (*Lavandula angustifolia* Oil) from lavender grown in the gardens of the Institute of Essential Oil of the Ukrainian Academy of Agricultural Sciences in Simferopol, Crimea, Ukraine. Both oils were obtained by steam distillation.

### 4.2. Gas Chromatography with Mass Spectrometry (GC–MS)

The analysis of Crimean lavender oil was performed at the Institute of Heavy Organic Synthesis “Blachownia” in Kędzierzyn-Koźle on an Agilent Technologies Gas Chromatograph 7890 GC (Agilent, Santa Clara, CA, USA) system coupled with a mass spectrometer, GC–MS 7000—Triple Quad (Agilent, Santa Clara, CA, USA). Two types of capillary columns of different polarity—non-polar HP-5MS (5% diphenyl, 95% dimethylpolysiloxane, Agilent J&W, Palo Alto, CA, USA) and SupelcoWAX^TM^ 10 polar (polyethylene glycol Carbowax® 20M, Merck KGaA, Darmstadt, Germany)—were employed; both columns had a length of 30 m, internal diameter of 0.25 mm, and film thickness of 0.25 microns. Helium served as the carrier gas, and its flow rate was 1.5 mL/min. Analyses were performed in the temperature range 45–250 °C; the initial temperature was maintained for 6 min, and the heating rate was 3 °C/min. Samples with a volume of 0.5 mL were prepared by means of an auto-sampler. The gas chromatograph was equipped with a split injector; the split ratio was 100:1. Injector temperature was 250 °C. The test solutions were prepared by diluting an oil sample with *n*-hexane at a volume ratio of 1:10 or 1:100.

ETJA lavender oil was analysed in the Faculty of Chemistry, University of Opole, on a Hewlett Packard HP 6890 series GC system chromatograph (Hewlett Packard, Waldbronn, Germany), which was coupled with a Hewlett Packard 5973 mass selective detector (Hewlett Packard, Waldbronn, Germany). The chromatograph was equipped with the non-polar, high-temperature ZB-5HT capillary column (length, 30 m; inner diameter, 0.32 mm; film thickness, 0.25 μm, Phenomenex Inc., Torrance, CA, USA). Helium served as the carrier gas, and its flow rate was 2 mL/min. Assays were performed in the temperature range 60–280 °C, and the heating rate was 10 °C/min; the auxiliary temperature was 300 °C. Samples with a volume of 1 mL were manually dosed. The gas chromatograph was equipped with an on-column injector with programmable temperature (the same as the analysis temperature). The test solutions were prepared by diluting an oil sample with dichloromethane at a volume ratio of 1:10 or 1:100.

Components were identified by comparison of their mass spectra with the spectrometer database of the NIST 11 Library (National Institute of Standards and Technology, Gaithersburg, MD, USA) and by comparison of their retention index calculated against *n*-alkanes (C_9_–C_20_). Each chromatographic analysis was repeated three times. The average values of relative composition of essential oil (percentages) were calculated from the peak areas.

### 4.3. Biological Experiment

The object of this experiment was the microbiota of oily facial skin without signs of lesions; the microbiota was isolated by a surface swab, and two lavender oils of various origins—from the ETJA company (ETJA, Elbląg, Poland) and oil extracted from Crimean lavender (not yet described in the literature)—were employed at concentrations 10–80 μL/cm^3^.

The biological material was collected from five areas of facial skin, i.e., the cheeks, nose, forehead, and chin (i.e., from a total area of 20 cm^2^) and was resuspended in broth (control) and in broth with the addition of tested lavender oils at concentrations of 20, 50, and 70 μL/cm^3^ (a concentration of 50 μL/cm^3^ is recommended by the manufacturers when this oil serves as an additive in cosmetic and pharmaceutical preparations). Because the effectiveness of an oil in suppressing the growth of the skin microbiota depends on the concentration and origin of the oil, lower and higher concentrations than those recommended by the manufacturer were tested. The samples were incubated for 24 h at a temperature of 35 °C. 

The anti-microbial effects of these oils on the mixed microbiota from facial skin were evaluated by the surface culture method (10-fold dilutions in water containing 0.05% Tween-80) in parallel with the Nutrient LAB Agar^TM^ medium by the BIOCORP company (BIOCORP, Warszawa, Poland) for determination of the bacterial cell count and in addition to selective media (Braid–Parker, *Enterococcus* agar, Hektoena, ENDO, and *Pseudomonas* agar) of the BTL company (BTL sp. z o.o., Łódź, Poland) for determination of a cell count of potentially pathogenic bacteria. After incubation, the total number of lavender oil-non-sensitive bacteria was determined, and the results were expressed in log colony-forming units (CFU)/cm^2^ of the facial skin surface. The dominant bacterial isolates were identified by API tests from BIOMERIEUX company (BIOMERIEUX SSC Europe Sp. z o.o., Warszawa, Poland; ID32GN: Gram-negative bacilli, 50CHB: Gram-positive bacilli, ID32 STAPH: Gram-positive cocci). In the presence of the highest concentration of ETJA lavender oil used, bacteria of the following species survived: *Micrococcus luteus*, *Escherichia coli*, *Staphylococcus warneri*, and *Enterococcus faecium*. In the presence of the highest concentration of Crimea oil used, bacteria of the following species survived: *Bacillus cereus*, *B. subtilis*, *B. mycoides*, *Corynebacterium* spp., and *Enterococcus faecium*.

Next, the bactericidal activities of the tested oils on these dominant bacterial isolates were evaluated by the diffusion cylinder plate method on Nutrient LAB Agar^TM^ medium [43]. The media were inoculated with 1 cm^3^ of a standard bacterial suspension with the optical density of ζ = 2 at a wavelength of 550 nm. The results were presented as a mean value of the growth inhibition diameter (in mm). The inhibitory effect was assumed to be the lack of growth around wells, whereas growth stimulation intensified growth around wells, and the neutral effect caused growth inhibition at the edges of the wells. The control was water containing 0.05% Tween-80. The essential oils and extracts were used at the following concentrations: 10, 20, 30, 40, 60, 70, or 80 μL/cm^3^ (*v*/*v*). Each experiment was repeated three times.

## 5. Conclusions

Lavender oils from ETJA and Crimea most effectively reduced the number of mixed microbiota cells from facial skin at a concentration of 70 µL/cm^3^, although no complete bactericidal activity was observed. The most sensitive to ETJA lavender oil were Gram-positive bacilli, and Gram-negative bacilli were the most sensitive to Crimean lavender oil. On the other hand, neither of the tested oils inhibited the growth of Gram-positive cocci. The tested lavender oils differed in their chemical composition quantitatively and qualitatively; 33 ingredients were identified in ETJA oil, including five compounds not described before (e.g., cyclofenchene and isoborneol); 101 components were identified in Crimean lavender oil, including 37 compounds not described before (e.g., octen-1-ol acetate and linalool formate). Two types of columns of different polarity allowed for better separation and identification of essential oil components such as limonene and eucalyptol. ETJA lavender oil was composed mainly of monoterpenoids (76.7%) and monoterpenes (22.7%), whereas Crimean lavender oil consisted mainly of monoterpenoids (80%), much less monoterpenes (5.8%), and some sesquiterpenes (8.0%; Table 5). Such differences in chemical composition were most likely due to the different geographical origins of the plant material. The analysed lavender oils differed in their bactericidal effect; ETJA lavender oil with higher monoterpenoid content (linalool and linalyl acetate) and monoterpene content (limonene) was characterised by higher efficiency than Crimean lavender oil.

## Figures and Tables

**Table 1:** The components of lavender oil from *Lavandula angustifolia* described in the literature.

Name	*Lavandula angustifolia*—Place and Area (%)								
	Italy			Australia		China	Greece	Brazil	Iran
	(a)	(b)	(c)	(a)	(b)		(a)		
tricyclene			-	0.02–0.04	-	-	-	-	-
*α*-thujene	0.13	0.11	0.05	0.02–0.17	-	-	-	-	-
*α*-pinene	0.51	0.93	0.26	0.08–0.73	-	-	-	0.70	1.41
camphene	0.26	0.59	0.19	0.02–0.54	0.19	3.98	-	0.50	-
*β*-phellandrene	-	-	-	0.02–0.11	-	-	-	3.40	-
*β*-pinene	0.14	0.30	1.58	0.03–1.21	-	-	-	1.10	1.52
octen-3-ol	0.22	0.20	0.16	0.03–1.14	0.28	0.35	-	-	-
3-octanone	0.36	0.68	-	0.33–3.49	-	-	-	-	-
myrcene	0.12	-	trace	0.26–1.22	0.56	0.87	-	0.70	1.02
3-carene	-	-	-	0.05–0.30	-	0.45	-	0.90	0.76
sabinene	0.31	0.75	0.31	0.04–0.39	-	-	-	-	-
*p*-cymene	0.29	0.20	-	0.10–0.45	-	-	-	-	-
*o*-cymene	0.04	0.09	0.30	0.03–0.12	-	-	-	-	-
limonene	1.10	2.36	-	0.18–3.92	0.24	0.19	-	-	-
eucalyptol	3.98	10.89	6.75	0.1–10.87	1.51	2.30	4.80	7.90	3.93
(*E*)-*β*-ocimene	1.24	0.42	0.56	0.34–2.36	0.09	0.52	-	-	-
(*Z*)-*β*-ocimene	1.02	1.32	0.77	0.95–6.17	0.44		-	-	-
*trans*-linalool oxide	-	0.07	1.57	0.26–0.99	-	-	-	-	-
*cis*-linalool oxide	0.09	0.07	1.48	0.34–1.09	0.24	-	-	-	1.67
*trans*-sabinene hydrate	0.39	0.34	-	0.04–0.20	-	-	-	-	-
linalool	35.96	36.51	35.31	23.03–57.48	52.59	44.54	44.50	-	4.91
*cis*-*p*-menth-2-en-1-ol	-	-	-	0.02–0.04	-	-	-	-	-
*trans*-pinocarveol	0.18	0.10	-	0.01–0.34	-	-	-	-	-
camphor	5.56	11.76	7.81	0.09–7.10	8.79	-	-	3.50	2.83
myrtenol	-	-	-	0.04–0.21	-	-	-	0.40	-
borneol	2.71	4.21	2.98	0.30–4.04	7.50	2.45	3.90	22.40	8.57
*p*-cymen-8-ol	0.33	0.55	-	0.12–0.27	-	-	-	-	-
lavendulol	0.05	0.05	0.55	0.05–0.86	-	-	-	-	-
terpinen-4-ol	6.57	2.10	3.34	0.11–8.07	2.45	-	6.90	0.90	-
sabine ketone	-	-	-	0.02–0.10	-	-	-	-	-
*m*-cymen-8-ol	0.03	0.09	-	0.02–0.18	-	-	-	-	-
*α*-terpineol	0.06	0.07	4.39	0.12–6.02	3.03	6.75	3.50	1.20	1.98
hexyl butyrate	-	-	0.43	0.12–1.72	-	-	-	-	-
isobornyl formate	0.06	0.10	-	0.10–0.52	-	-	-	-	-
geraniol	-	-	0.67	-	-	11.02	-	-	1.24
cumin aldehyde	-	-	-	0.04–0.53	0.16	-	-	-	-
carvone	-	-	-	0.02–0.19	-	-	-	0.40	-
linalyl acetate	21.74	14.42	12.09	4.01–35.39	9.27	-	32.70	-	-
*α*-bisabolol	1.12	0.89	3.76	0.02–0.71	0.78	-	-	13.10	2.32
dihydrocarveol	-	-	-	0.03–0.51	-	-	-	-	-
bornyl acetate	-	-	-	0.03–0.32	1.11	-	-	-	1.67
lavendulyl acetate	-	-	-	0.70–6.16	1.32	10.78	-	-	-
neryl acetate	0.06	-	1.31	0.07–1.23	1.21	-	-	-	2.16
*β*-bourbonene	0.17	0.09	-	0.02–0.09	-	-	-	-	-
*α-trans*-bergamotene	0.07	0.05	-	0.02–0.15	0.07	-	-	-	-
*α*-cederene	-	-	-	0.01–0.09	-	-	-	-	-
*β*-caryophellene	-	-	-	-	-	-	-	3.20	1.60
caryophyllene	2.87	2.42	1.30	0.45–2.83	1.00	0.50	0.30	-	-
*α*-santalene	-	-	-	-	0.05	-	-	-	-
*α-cis*-bergamotene	0.07	0.06	-	0.02–0.09	-	-	-	-	-
*β*-farnesene	4.02	1.07	1.00	0.17–1.69	1.83	-	-	-	0.71
germacene D	0.77	1.50	-	0.16–0.94	0.37	-	-	-	-
*β*-bisabolene	0.03	-	-	-	0.26	-	-	0.80	-
*γ*-cadinene	0.26	0.39	-	0.03–0.39	0.28	-	-	2.90	-
caryophyllene oxide	-	-	-	-	0.22	-	-	4.50	2.73
spathulenol	0.06	0.31	-	0.01–0. 06	-	-	-	-	-
*γ*-muurolol	-	-	-	0.02–0.48	-	-	-	-	-
*τ*-cadinol	-	-	-	0.02–0.42	-	-	-	-	1.85
*γ*-terpinene	-	-	-	0.05–0.22	-	-	-	-	-
octen-3-yl acetate	-	-	-	0.19–4.16	-	-	-	-	-
norborneol acetate	-	-	-	0.04–0.51	-	-	-	-	-

Italy (a)—low Friuli–Venezia Giulia (northeast Italy) [7]; Italy (b)—high Friuli–Venezia Giulia (northeast Italy) [7]; Italy (c)—Betulla srl (Italy) [9]; Australia (a)—Australian Botanical Products (Hallam, Australia) [13]; Australia (b)—The Lavender Patch lavender farm, Victoria, Australia [8]; China (a)—Xinjiang, China [14]; Greece (a)—Crete (Greece) [15]; Brazil—Franca, State of Sao Paulo, Brazil [10]; Iran—Astara, north of Iran [16].

**Table 2:** The components of other lavender oils described in the literature.

Name	Place and Area (%) ± SD								
	*Lavandin*	*Lavandula*							
	*abrialis*	*stoechas*		*dentata*	*bipinnata*	*gibsoni*	*canarien-sis*	*multifida*	
	France	Turkey	Greece	Algeria	India	India	Australia	Portugal	
			(b)		(a)	(b)	(c)	(a)	(b)
tricyclene	0.03	-	0.20	0.40	-	-	-	-	-
*α*-thujene	-	-	-	trace	-	-	-	-	-
*α*-pinene	0.40	1.31	2.52	trace	1.01	1.47	trace	0.80	0.60
camphene	0.30	1.40	1.30	-	0.35	-	-	-	-
*β*-phellandrene	-	-	0.10	-	-	-	-	-	-
*β*-pinene	0.30	-	1.30	0.20	-	-	trace	-	-
octen-3-ol	0.30	-	-	-	-	2.20	-	0.60	0.50
3-octanone	1.00	-	-	-	-	-	-	0.40	0.30
myrcene	0.30	-	0.10	trace	0.25	2.78	trace	5.70	5.50
3-carene	0.02	-	-	-	0.37	1.52	-	0.50	0.50
sabinene	0.10	-	-	1.40	0.39	-	-	-	-
*p*-cymene	0.04	-	4.90	-	-	0.82	trace	0.30	0.20
*o*-cymene	-	-	-	-	-	-	-	-	-
limonene	0.70	-	-	-	-	2.30	trace	0.60	0.30
eucalyptol	-	8.03	16.30	38.40	-	-	-	-	-
(*E*)-*β*-ocimene	2.60	-	trace	0.10	-	trace	trace	27.40	27.00
(*Z*)-*β*-ocimene	3.00	-	-	-	-	0.30	0.60	1.70	1.50
*trans*-linalool oxide	0.20	-	-	-	-	-	-	-	-
*β*-citral						-	0.30	-	-
*cis*-linalool oxide	0.1	-	-	-	-	-	-	-	-
*trans*-sabinene hydrate	-	-	-	0.10	-	-	-	-	-
linalool	35.00	0.29	1.20	trace	0.94	2.65	0.90	0.30	0.20
*cis*-*p*-menth-2-en-1-ol	-	-	-	trace	-	-	-	-	-
*trans*-pinocarveol	-	-	-	-	-	-	-	-	-
camphor	8.90	18.18	9.90	1.60	7.09	-	-	-	-
myrtenol	-	-	1.10	1.70	-	-	-	-	-
borneol	2.90	-	0.90	-	-	-	-	-	-
*p*-cymen-8-ol	-	-	-	3.80	-	-	0.20	-	-
lavandulol	0.60	-	0.40	-	0.38	-	-	-	-
terpinen-4-ol	-	-	0.80	0.70	0.73	-	-	-	-
sabine ketone	-	-	-	0.50	-	-	-	-	-
*m*-cymen-8-ol	-	-	0.20	-	-	-	-	-	-
*α*-terpineol	0.50	-	0.50	1.80	-	0.77	0.30	-	-
hexyl butyrate	-	-	-	-	-	-	-	-	-
isobornyl formate	-	-	-	-	-	-	-	-	-
geraniol	-	-	-	-	-	-	-	-	-
cumin aldehyde	-	-	-	1.10	-	-	-	-	-
carvone	-	-	0.10	-	-	-	-	-	-
linalyl acetate	27.00	-	0.20	trace	3.37	-	-	-	-
*α*-bisabolol	-	-	trace	-	-	-	1.50	0.20	0.20
dihydrocarveol	-	-	-	-	-	-	-	-	-
bornyl acetate	-	1.32	trace	-	0.21	-	-	-	-
lavendulyl acetate	1.00	-	3.21	-	1.79	-	-	-	-
verbenone	-	-	0.60	0.40	-	-	-	-	-
neryl acetate	0.70	-	-	-	-	-	-	-	-
*β*-bourbonene	-	-	-	trace	-	-	trace	-	-
*α-trans*-bergamotene	-	-	-	trace	-	-	-	-	-
*α*-cederene	-	-	-	trace	-	-	-	-	-
*β*-caryophellene	-	-	-	-	0.18	-	7.60	0.80	0.90
caryophyllene	0.70	-	-	0.50	-	trace	-	-	-
*α*-santalene	0.20	-	-	-	-	-	-	-	-
*α-cis*-bergamotene	-	-	-	-	-	-	-	-	-
*β*-farnesene	0.30	-	-	-	-	-	0.30	trace	0.10
germacene D	-	-	0.10	-	1.66	-	2.20	0.50	0.30
*β*-bisabolene	-	-				-	20.80	5.60	5.00
*γ*-cadinene	trace	0.80	0.20	0.40	-	-	-	0.20	0.20
caryophyllene oxide	0.30	0.33	0.90	-	3.68	1.21	2.00	0.30	0.20
spathulenol	-	-	-	0.30	-	-	2.20	0.60	0.80
cubenol	-	-	trace	-	-	-	-	-	-
*γ*-muurolol	-	-	-	-	-	-	-	-	-
*τ*-cadinol	-	-	4.20	-	-	-	-	0.20	0.30
*γ*-terpinene	trace	-	0.20	0.50	-	-	-	0.20	0.10
octen-3-yl acetate	0.03	-	-	-	-	-	-	-	-
norborneol acetate	-	-	-	-	-	-	-	-	-

France—southern France [11]; Turkey—district of Alahan (Hatay) [17]; Greece (b)—north part of Greece at Chalkidiki peninsula [18]; Algeria—Cherchel (northwest of Algiers region, Algeria) [12]; India (a)—Asangihal village in Sindagi taluk of Bijapur district, India [19]; India (b)—Purandar Fort region [20]; Australia (c)—Randwick, Sydney (Australia) [21]; Portugal (a)—region Sesimbra/Arrábida, south of Portugal [22]; Portugal (b)—region Mértola, south of Portugal [17].

**Table 3:** The components of ETJA and Crimean lavender oils.

Name	Abbreviation	RI		Oil, Column and Area (%) ± SD		
		Literat.	Exper.	ETJA	Crimean	
				ZB-5HT	HP-5MS	SupelcoWAX
tricyclene	B MO	923	920	-	0.04 ± 0.00	0.01 ± 0.00
*α*-thujene	B M	928	928	-	0.18 ± 0.01	0.04 ± 0.00
*α*-pinene	B M	936	933	0.7 ± 0.01	0.36 ± 0.01	-
camphene	B M	950	947	0.31 ± 0.02	0.27 ± 0.01	0.05 ± 0.01
*β*-phellandrene	M M	973	973	0.09 ± 0.00	0.09 ± 0.01	-
*β*-pinene	B M	978	974	0.22 ± 0.01	0.11 ± 0.01	-
octen-3-ol	OT	980	983	0.04 ± 0.01	0.61 ± 0.01	0.16 ± 0.01
3-octanone	OT	985	988	-	0.10 ± 0.01	0.01 ± 0.01
*β*-myrcene	A M	989	991	0.38 ± 0.02	0.25 ± 0.01	0.08 ± 0.00
3-carene	B M	1011	1005	0.36 ± 0.01	0.86 ± 0.01	0.20 ± 0.01
sabinene	B M	1004	1009	-	0.02 ± 0.01	0.02 ± 0.01
*p*-cymene	M M	1024	1020	0.58 ± 0.01	0.22 ± 0.02	-
*o*-cymene	M M	1041	1022		1.03 ± 0.06	0.18 ± 0.00
limonene	M M	1029	1026	19.02 ± 0.07	0.55 ± 0.02	0.15 ± 0.00
eucalyptol	B MO	1031	1027	-	5.00 ± 0.10	1.66 ± 0.02
(*E*)-*β*-ocimene	A M	1048	1040	0.02 ± 0.01	0.99 ± 0.01	0.44 ± 0.01
(*Z*)-*β*-ocimene	A M	1037	1049		0.75 ± 0.02	0.41 ± 0.01
*trans*-linalool oxide	A MO	1083	1070	0.05 ± 0.01	0.53 ± 0.03	0.08 ± 0.00
*β*-citral	A M	1245	1084	-	0.08 ± 0.01	-
*cis*-linalool oxide	A MO	1075	1091	0.15 ± 0.01	0.52 ± 0.02	0.07 ± 0.01
*trans*-sabinene hydrate	B MO	1098	1097	-	0.10 ± 0.04	0.04 ± 0.00
linalool	A MO	1099	1105	41.84 ± 0.10	34.13 ± 0.25	52.71 ± 0.33
*cis*-*p*-menth-2-en-1-ol	M MO	1123	1116	-	0.01 ± 0.00	-
*trans*-pinocarveol	B MO	1140	1135	-	0.04 ± 0.01	-
camphor	B MO	1143	1141	0.15 ± 0.01	0.54 ± 0.01	0.09 ± 0.01
myrtenol	B MO	1150	1146	0.19 ± 0.01	0.08 ± 0.04	-
borneol	B MO	1166	1168	-	1.50 ± 0.01	-
*p*-cymen-8-ol	B MO	1184	1171	-	0.04 ± 0.01	0.03 ± 0.00
lavandulol	A MO	1168	1175	0.18 ± 0.01	0.54 ± 0.01	0.11 ± 0.02
terpinen-4-ol	M MO	1177	1181	0.29 ± 0.01	6,66 ± 0.04	2.29 ± 0.02
sabine ketone	B MO	1194	1190	-	0.50 ± 0.02	0.08 ± 0.00
*m*-cymen-8-ol	B MO	1180	1192	0.03 ± 0.01	0.16 ± 0.01	0.03 ± 0.00
*α*-terpineol	M MO	1190	1197	0.07 ± 0.00	1.54 ± 0.03	-
hexyl butyrate	OT	1191	1203	-	0.63 ± 0.02	-
isobornyl formate	B MO	1240	1230	-	0.13 ± 0.01	0.61 ± 0.01
geraniol	A MO	1255	1234	0.02 ± 0.01	0.08 ± 0.01	0.03 ± 0.01
cumin aldehyde	M MO	1238	1242	-	0.18 ± 0.00	0.03 ± 0.00
carvone	M MO	1242	1245	0.04 ± 0.01	0.05 ± 0.01	0.01 ± 0.00
linalyl acetate	A MO	1255	1259	32.70 ± 0.08	23.29 ± 0.30	36.56 ± 0.34
*α*-bisabolol	M SO	1282	1266	-	0.03 ± 0.01	-
dihydrocarveol	M MO	1194	1277	-	0.05 ± 0.07	-
bornyl acetate	B MO	1283	1278	-	0.15 ± 0.01	0.04 ± 0.01
lavendulyl acetate	A MO	1289	1285	0.06 ± 0.01	2.45 ± 0.02	0.51 ± 0.01
verbenone	B MO	1206	1296	-	0.04 ± 0.01	-
neryl acetate	A MO	1363	1359	0.39 ± 0.02	0.22 ± 0.01	0.05 ± 0.01
*β*-bourbonene	B S	1384	1373	-	0.05 ± 0.00	0.01 ± 0.00
*α-trans*-bergamotene	B S	1434	1382	-	0.17 ± 0.01	-
*α*-cedrene	B S	1412	1398	-	0.06 ± 0.00	-
*β*-caryophellene	B S	1406	1402	-	0.11 ± 0.10	-
caryophyllene	B S	1420	1408	0.5 ± 0.01	4.19 ± 0.10	1.63 ± 0.02
*α*-santalene	B S	1421	1411	-	1.05 ± 0.03	0.23 ± 0.01
*α-cis*-bergamotene	B S	1414	1429	-	0.28 ± 0.00	0.02 ± 0.00
*β*-farnesene	A S	1456	1454	0.1 ± 0.01	0.96 ± 0.01	0.28 ± 0.02
germacene D	A S	1481	1496	-	0.03 ± 0.02	-
*β*-bisabolene	M S	1508	1504	-	0,02 ± 0.01	-
*γ*-cadinene	B S	1513	1507	-	0.53 ± 0.01	-
caryophyllene oxide	B SO	1581	1572	-	2.59 ± 0.01	0.29 ± 0.00
spathulenol	B SO	1576	1601	-	0.02 ± 0.00	-
cubenol	B SO	1636	1603	-	0.04 ± 0.01	-
*γ*-muurolol	B S	1645	1622	-	0.02 ± 0.01	0.07 ± 0.00
*τ*-cadinol	B SO	1640	1628	-	0.25 ± 0.01	0.03 ± 0.01
*γ*-terpinene	M M	1060	1060	0.08 ± 0.01	-	0.02 ± 0.00
octen-3-yl acetate	OT	1110	1091	-	-	0.21 ± 0.01
norborneol acetate	B MO	1114	1129	-	-	0.08 ± 0.00
*α*-terpinene	M M	1017	1016	0.53 ± 0.01	-	-
Not described in literature						
*m*-cymene	M M	999	970	-	0.03 ± 0.01	-
butanoic acid, butyl ester	OT	990	997	-	0.15 ± 0.01	0.13 ± 0.01
acetic acid, hexyl ester	OT	1004	1016	-	0.22 ± 0.00	0.07 ± 0.00
bicyclo[3.1.0]hexan-2-ol	B MO		1064	-	0.20 ± 0.01	-
isopulegone	M MO	1157	1068	-	0.01 ± 0.00	-
eucarvone	M MO	1048	1077	-	0.01 ± 0.00	-
2-carene	B M	1006	1079	0.02 ± 0.0	0.01 ± 0.01	-
6-camphenol	B MO	1110	1081	-	0.01 ± 0.00	-
cinerone	M MO	1084	1088	-	0.04 ± 0.01	-
octen-1-ol acetate	OT	1192	1112	-	1.06 ± 0.04	-
isopulegol isomer	M MO	1146	1139	-	0.03 ± 0.01	-
butanoic acid, hexyl ester	OT	1190	1156	-	0.11 ± 0.01	0.02 ± 0.01
*Z*-farnesol	A SO		1160	-	0.02 ± 0.01	-
3,7 octadiene-2,6 diol 2,6-dimethyl isomer	A MO	1189	1200	-	0.22 ± 0.01	0.02 ± 0.00
isopulegol isomer	M MO	1156	1214	-	0.02 ± 0.01	-
*p*-menthane-1,2,3-triol	M MO		1251	-	0.16 ± 0.01	-
*E*-farnesol	A SO		1269	-	0.02 ± 0.01	-
α-limonene diepoxide	M MO	1297	1271	-	0.13 ± 0.01	-
3,7-octadiene-2,6-diol-2,6-dimethyl isomer	A MO	1229	1332	-	0.12 ± 0.01	-
hydroxy linalool	A MO	1367	1345	-	0.15 ± 0.01	-
limonene oxide	M MO	1336	1348	-	0.15 ± 0.00	-
epicubenol	B SO	1627	1364	-	0.03 ± 0.00	-
linalool formate	A MO	1219	1379	-	0.27 ± 0.01	-
nerolidyl acetate	A SO	1687	1385	-	0.01 ± 0.00	-
*β*-cedrene	B S	1419	1439	-	0.09 ± 0.01	-
epi-*β*-santalene	B S	1431	1441	-	0.04 ± 0.01	0.01 ± 0.00
santalol	B SO	1617	1442	-	0.01 ± 0.00	-
humulene	B S	1455	1444	-	0.13 ± 0.01	0.03 ± 0.00
limonen-6-ol pivalate	M SO		1452	-	0.13 ± 0.01	-
*β*-cubenene	B S	1527	1473	-	0.09 ± 0.00	
*β*-carryophyllene isomer	B S	1455	1479	-	0.10 ± 0.02	-
*α*-santanol	B SO	1671	1514	-	0.06 ± 0.01	-
calamenene	B S	1543	1515	-	0.03 ± 0.01	-
tricyclo[7.2.0.0(2,6)]undecan-5-ol,2,6,10-tetramethyl	B SO		1548	-	0.07 ± 0.01	-
zingiberene	M S	1495	1495	-	-	0.01 ± 0.00
benzeneethanol	OT	1121	1120	-		0.06 ± 0.01
cyclofenchene	B M	882	892	0.34 ± 0.01	-	-
*β*-copaene	B M	1433	1433	0.04 ± 0.01	-	-
2-bornanone	B MO	1143	1136	0.05 ± 0.01	-	-
isoborneol	B MO	1158	1145	0.46 ± 0.01	-	-

Abbreviations: A M—aliphatic monoterpenes; M M—monocyclic monoterpenes; B M—bi- and tricyclic monoterpenes; A MO—aliphatic monoterpenoids; M MO—monocyclic monoterpenoids; B MO—bi- and tricyclic monoterpenoids; A S—aliphatic sesquiterpenes; M S—monocyclic sesquiterpenes; B S—bi- and tricyclic sesquiterpenes; A SO—aliphatic sesquiterpenoids; M SO—monocyclic sesquiterpenoids; B SO—bi- and tricyclic sesquiterpenoids. SD—standard deviation; RI—retention indexes; literat.—literature data; exper.—determined experimentally for the non-polar columns: HP-5MS for CRIMEA oil and ZB-5HT for ETJA oil.

**Table 4:** The list of terpenes in the tested lavender oils.

	Abbreviation	Oil, Column and Area (%)		
		ETJA	Crimean	
		ZB-5HT	HP-5MS	SupelcoWAX
Aliphatic monoterpenes	A M	0.40	2.07	0.93
Monocyclic monoterpenes	M M	20.30	1.92	0.35
Bi- and tricyclic monoterpenes	B M	1.99	1.81	0.31
**Monoterpenes**	**M**	**22.69**	**5.80**	**1.59**
Aliphatic monoterpenoids	A MO	75.39	62.52	90.14
Monocyclic monoterpenoids	M MO	0.40	9.04	2.33
Bi- and tricyclic monoterpenoids	B MO	0.88	8.53	2.67
**Monoterpenoids**	**MO**	**76.67**	**80.09**	**95.14**
Aliphatic sesquiterpenes	A S	0.10	0.99	0.28
Monocyclic sesquiterpenes	M S	-	0.02	0.01
Bi- and tricyclic sesquiterpenes	B S	0.50	6.94	2.00
**Sesquiterpenes**	**S**	**0.60**	**7.95**	**2.29**
Aliphatic sesquiterpenoids	A SO	-	0.05	-
Monocyclic sesquiterpenoids	M SO	-	0.16	-
Bi- and tricyclic sesquiterpenoids	B SO	-	3.07	0.32
**Sesquiterpenoids**	**SO**	**-**	**3.28**	**0.32**
**Others**	**OT**	**0.04**	**2.88**	**0.66**

**Table 5:** Zones of growth inhibition of dominant bacterial isolates.

Species of Isolates	Oil Concentration (μL/cm^3^)	Zones of Inhibition (mm) ± SD	
		ETJA	Crimean
*Bacillus cereus*	40	47.0 ± 4.2	0
	80	40.0 ± 3.5	19.5 ± 0.7
*Bacillus* subtilis	80	23.5 ± 0.7	0
*Bacillus mycoides*	60	33.0 ± 1.4	0
*Staphylococcus warneri*	80	22.8 ± 0.4	0
*Micrococcus luteus*	80	19.0 ± 1.4	12.5 ± 0.7
*Enterococcus faecium*	80	0	0
*Corynebacterium* spp.	50	18.5 ± 0.7	13.0 ± 1.4
*Escherichia coli*	80	0	16.0 ± 1.4
